# The Role of Acetyl Zingerone and Its Derivatives in Inhibiting UV-Induced, Incident, and Delayed Cyclobutane Pyrimidine Dimers

**DOI:** 10.3390/antiox12020278

**Published:** 2023-01-26

**Authors:** Jyoti Srivastava, Montana M. Young, Vipin Kumar Yadav, Pravin R. Phadatare, Thomas A. Meyer, Ratan K. Chaudhuri, Sanjay Premi

**Affiliations:** 1 Tumor Biology, Moffitt Cancer Center, 12902 USF Magnolia Drive, Tampa, FL 33612, USA; 2 SYTHEON, 10 Waterview Blvd, Parsippany, NJ 07054, USA

**Keywords:** melanin chemiexcitation (MeCh), melanin synthesis, cyclobutane pyrimidine dimers (CPDs), incident and delayed CPDs, nucleotide excision repair

## Abstract

Cyclobutane pyrimidine dimers (CPDs) are ultraviolet radiation (UV)-induced carcinogenic DNA photoproducts that lead to UV signature mutations in melanoma. Previously, we discovered that, in addition to their incident formation (iCPDs), UV exposure induces melanin chemiexcitation (MeCh), where UV generates peroxynitrite (ONOO^−^), which oxidizes melanin into melanin-carbonyls (MCs) in their excited triplet state. Chronic MeCh and energy transfer by MCs to DNA generates CPDs for several hours after UV exposure ends (dark CPD, dCPDs). We hypothesized that MeCh and the resulting dCPDs can be inhibited using MeCh inhibitors, and MC and ONOO^−^ scavengers. Here, we investigated the efficacy of Acetyl Zingerone (AZ), a plant-based phenolic alkanone, and its chemical analogs in inhibiting iCPDs and dCPDs in skin fibroblasts, keratinocytes, and isogenic pigmented and albino melanocytes. While AZ and its methoxy analog, 3-(4-Methoxy-benzyl)-Pentane-2,4-dione (MBPD) completely inhibited the dCPDs, MBPD also inhibited ~50% of iCPDs. This suggests the inhibition of ~80% of total CPDs at any time point post UV exposure by MBPD, which is markedly significant. MBPD downregulated melanin synthesis, which is indispensable for dCPD generation, but this did not occur with AZ. Meanwhile, AZ and MBPD both upregulated the expression of nucleotide excision repair (NER) pathways genes including *Xpa*, *Xpc*, and *Mitf*. AZ and its analogs were non-toxic to the skin cells and did not act as photosensitizers. We propose that AZ and MBPD represent “next-generation skin care additives” that are safe and effective for use not only in sunscreens but also in other specialized clinical applications owing to their extremely high efficacy in blocking both iCPDs and dCPDs.

## 1. Introduction

In the USA, ~9000 people are diagnosed with skin cancers every day, with more than two deaths occurring every hour [1,2]. Sunburns and chronic sun exposure double the risk of skin cancers [2], mostly through bulky and extremely mutagenic DNA photoproducts called cyclobutane pyrimidine dimers (CPDs). CPDs lead to cytosine to thymine (C to T) transition mutations, also known as UV signature mutations [3,4]. CPDs are generated in picoseconds in response to UV exposure (incident or iCPDs). The pigment melanin, produced by melanocytes, is known to act as a sunshield owing to its abnormally broad absorption spectrum and partial antioxidant nature [5]. Two subtypes of melanoma, eumelanin and pheomelanin, have different capabilities with regard to protecting the skin. Eumelanin is dark/black in color and a potent sunshield and antioxidant [6]. Pheomelanin is less photostable, and more oxidative compared to eumelanin, which is added upon by the fact its synthesis consumes cysteine, an integral part of the cellular antioxidant glutathione [6,7]. Contrary to its known photoprotective properties, Dr. Brash and his team discovered a novel pathway, called melanin chemiexcitation (MeCh) that implicates melanin in the generation of CPDs in complete absence of UV radiation [8]. In MeCh, UV exposure induces nitric oxide synthase (NOS) and NADPH oxidase (NOX) activities to produce peroxynitrite (ONOO^−^), which subsequently reacts with melanin to generate triplet-excited melanin-carbonyls (MCs). These MCs, which are as energetic as UV photons, can transfer their energy to di-pyrimidines, generating CPDs without UV exposure (delayed or dark CPDs, dCPDs). At any time-point post UV exposure, approximately 50% of CPDs are generated by MeCh [8,9] suggesting a potentially carcinogenic role for melanin. We also demonstrated that MeCh might be operative without any UV exposure, due only to endogenously hyperactive NOS in pigmented melanocytes [8]. Pursuing this further, we identified genomic sites that are hypersensitive to iCPDs and dCPDs [9]. These findings suggest a carcinogenic role for melanin. This also indicates that the total amount of CPDs generated by UV exposure has always been underestimated, and so is the mutational load in sunlight-induced skin cancers [9].

Sunscreens significantly inhibit iCPD formation during sun exposure. Sunscreens form a continuous film that creates an effective interface between the sunlight and the skin. During sun exposure, sunscreen compounds are evenly dispersed within the film, and intercept and neutralize UV photons before they can reach and damage the underlying skin. Indeed, research has established that regular use of sunscreens does delay or reduce the incidences of melanoma and non-melanoma skin cancers [10,11]. Several mechanisms have been proposed for this [12,13]; however, the sunscreen-mediated inhibition of iCPD formation seems to be the best explanation. The fact that sunscreens reduce the total amount of CPDs significantly has been observed in both in situ and in vivo models [14,15,16,17,18]. 

While effective during periods of sun exposure, it should be noted that sunscreen compounds can also act as a photosensitizers [19] and can be cytotoxic [20,21,22,23]. Additionally, sunscreens are completely ineffective against dCPD formation once sun exposure ends. This is particularly important in the case of pigmented melanocytes, which have melanin as a raw material, in addition to nuclear and cytoplasmic NOS activity as an inducer of melanin chemiexcitation [8]. Previously, we demonstrated that a plant-based phenolic alkanone called 3-(4-hydroxy, 3-methoxybenzyl) -pentane-2,4-dione (INCI: acetyl zingerone (AZ)) effectively blocked melanin chemiexcitation and inhibited dCPD generation [24]. We postulated that AZ could intervene in the MeCh pathway, mainly by scavenging ONOO^−^ or by physically quenching the triplet energy of melanin-carbonyls via energy transfer [25]. Using expressional microarrays on reconstituted human epidermis, we also reported that AZ increased the expression of NOTCH pathway genes such as *NOTCH1* and *MAML3* and decreased the expression of genes linked to ECM disassembly (*MMP3*, *CTSV*, *NOXO1*) and reactive oxygen species metabolism (*PMAIP1*, *ARG2*). In in vitro assays, AZ also inhibited the activity *of* MMP-1, MMP-3, and MMP-12 and consistently increased the expression of genes encoding collagens, proteoglycans, ECM regulators, and ECM glycoproteins, while concomitantly opposing the gene expression patterns associated with fibroblast senescence, keratinocyte differentiation, and IL-17A stimulation [26].

**Figure 1:**
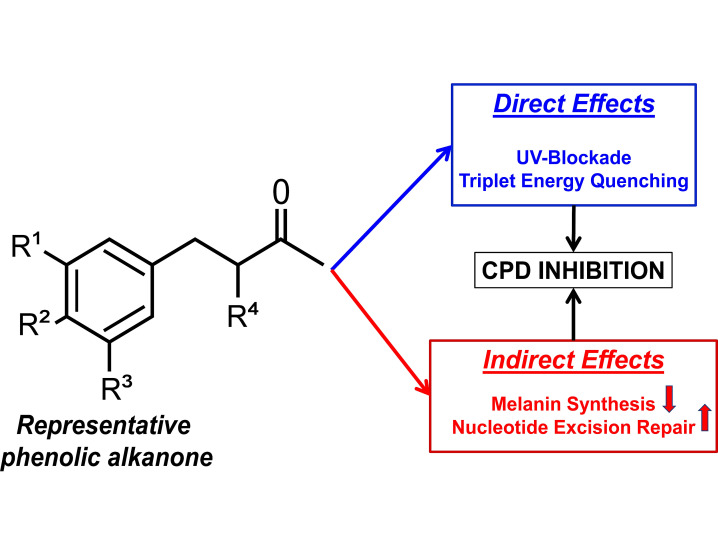
We investigated the potency of a plant-based, phenolic alkanone in blocking the harmful effects of ultraviolet (UV) radiation. The backbone structure of this alkanone is shown here with four “R” groups. Differing the functional groups at each “R” individually, we generated seven chemical analogs, including Acetyl Zingerone (AZ), described in the Methods section. AZ can scavenge peroxynitrite directly. Additionally, our unpublished data suggest that AZ and its analogs can potentially interfere with triplet–triplet and singlet–singlet energy transfers, thus preventing direct CPD formation. Indirectly, both AZ and MBPD upregulated the nucleotide excision repair (NER) pathway which exclusively repairs CPDs. Additionally, MBPD also reduced the amount of melanin present. Together, this led to the inhibition of melanin chemiexcitation, a pathway responsible for delayed formation of cyclobutane pyrimidine dimers (CPDs). Combined with the direct inhibition of CPDs that we observed, we propose this phenolic alkanone as a multifunctional, active ingredient with clinical relevance for use in sunscreens and other topical applications, since CPDs are highly mutagenic adducts and lead to >80% of mutations in skin cancers including melanoma. The up- and downward direction of arrows in “indirect effects” represent up- and downregulation, respectively, of the nucleotide excision repair and melanin synthesis pathways.

In the current study, we further evaluated AZ as well as six of its chemical analogs for their effectiveness in blocking both iCPD and dCPD formation and investigated their basic structure–activity requirements for this property alongside mechanisms of action. We investigated their cellular toxicity, effects on DNA repair, and efficiency in inhibiting CPD generation in response to exposure to solar-simulated UV (ssUV). We identified MBPD (3-(4-Methoxy-benzyl)-pentane-2,4-dione) as one analog which effectively inhibited ~80% of the total CPD formation (iCPDs + dCPDs) in pigmented mouse and human melanocytes. Taken together, our results strongly support the use of AZ and MBPD as skincare additives in sunscreens as well as other topically applied products to bolster protection against CPD formation. Figure 1 displays the general backbone of AZ’s chemical structure and summarizes the overall findings of this research. 

## 2. Materials and Methods

### 2.1. Acetyl Zingerone and Its Analogs

The chemical synthesis of Acetyl Zingerone has been described previously by us [24]. Briefly, vanillin (4-hydroxy-3-methoxybenzaldehyde) was condensed with acetylacetone to obtain 3-(4-hydroxy, 3-methoxybenzylidine) -pentane-2,4-dione, and the reaction intermediates were catalytically hydrogenated to obtain the target compound. The purity of AZ was estimated to be >99% as determined by HPLC. The structure was confirmed by ^1^HNMR, ^13^CNMR, and mass spectral analysis. Acetyl zingerone (trade name: Synoxyl^®^ AZ) is commercially available from Sytheon, Parsippany, New Jersey, USA. AZ is REACH-registered (EC# 820-605-0) and is safe for topical applications, as documented by the studies available for review on the ECHA website. The backbone structure is shown in Figure 1. The functional groups on all the “Rs” and the chemical names with their respective abbreviations are listed below.

- 3-(4-Hydroxy-3-methoxybenzyl)-pentane-2,4-dione [Acetyl Zingerone (Synoxyl^®^ AZ)] R^1^ = H, R^2^ = OH, R^3^ = OCH_3_, R^4^ = COCH_3_ (**AZ**). This is the parent compound which we have published previously for its capabilities in blocking delayed CPD generation [17].
- 2-Vanillyl-acetoacetic acid ethyl ester, R^1^ = H, R^2^ = OH, R^3^ = OCH_3_, R^4^ = COOCH_2_CH_3_ (**VAAE**).
- 3-(3,4,5-trimethoxy-benzyl)-pentane-2,4-dione, R^1^ = R^2^ = R^3^ = OCH_3_, R^4^ = COCH_3_, (**TMBPD**).
- 3-(4-Methoxy-benzyl)-pentane-2,4-dione, R^1^ = R^3^ = H, R^2^ = OCH_3_, R^4^ = COCH_3_ (**MBPD**).
- 3-(3,4-Dimethoxy-benzyl)-pentane-2,4-dione [Acetyl Zingerone Methyl Ether (Synoxyl^®^ MAZ)] R^1^ = H, R^2^ = R^3^ = OCH_3_, R^4^ = COCH_3_ (**DMBPD**).
- 4-(4-Hydroxy-3-methoxyphenyl)-butan-2-one [**Zingerone**]. *Purchased from Sigma-Aldrich, Cat. W312420*. R^1^ = H, R^2^ = OH, R^3^ = OCH_3_, R^4^ = H.
- 4-(4-Methoxyphenyl)-butan-2-one [Raspberry Ketone Methyl Ether **RKME**]. *Purchased from Sigma-Aldrich, Cat. W267201*. R^1^ = R^3^ = R^4^ = H, R^2^ = OCH_3_.

AZ and its analogs were dissolved in 200 mg/mL DMSO and diluted in cell culture medium at various concentrations for treating the cells in the culture. The respective molar concentrations are mentioned in the results and figure legends for each AZ analog. 

### 2.2. Cell Types and Source

Various human and mouse skin cells were used for testing the efficacy of AZ and its analogues as sunscreens and inhibitors of MeCh. This included primary human skin fibroblasts (NBHF); primary human skin melanocytes (PHM); pigmented, C57BL/6 mouse melanocytes (Mel-a); and tyrosinase mutated, isogenic albino melanocytes (Mel-c). All were purchased from the specimen resource core of the Dermatology Department, Yale School of Medicine, New Haven, under a material transfer agreement (MTA). Mel-a and Mel-c, originally called Melan-a and Melan-c, are immortalized, C57BL/6 melanocytes generated by Dr. Bennett’s group. Mel-a cells were generated from embryonic melanoblasts of inbred C57BL/6 mice [27]. Mel-c (albino melanocyte) cells were generated from the epidermis of C57BL/6 mice carrying a mutation in the albino locus which codes for tyrosinase [28]. HaCaT, human keratinocyte cells, were purchased from AddexBio, San Diego, CA, USA (Cat. T0020001). 

### 2.3. Cell Culture, Treatment of Cells with AZ and Its Analogs, and Cell Survival Assays

Mel-a and Mel-c melanocytes were cultured in a OptiMem base (Cat. 31985-070, Life Technologies, Carlsbad, CA, USA) supplemented with 7% horse serum (Cat. 100-508, Gemini Bio-Products, W. Sacramento, CA, USA), and 10 ng/mL (16.2 nM) TPA (12-O-tetradecanoylphorbol-13-acetate, Cat. P1585, Sigma, St. Louis, MO, USA) [8]. NBHF and HaCaT cells were cultured in high glucose DMEM supplemented with 5% FBS (GeminiBio, Cat. 100-500) [9]. Cell culture medium for every cell type was supplemented with 1× penicillin/streptomycin (diluted from 100×, which is 100 U/mL penicillin and 100 µg/mL streptomycin, Cat. 15140-122, Life Technologies, Carlsbad, CA, USA) throughout the experiment, and with Mycoplasma Removal Agent (Cat. BUF035, Bio-Rad Laboratories, Hercules, CA, USA) for the first 3 passages. Cells were regularly tested for Mycoplasma using PCR based methods (Cat. MP0025, Sigma). Cells were treated with various concentrations of AZ and its analogs in 96-well plates (Cat. 165305, ThermoFisher Scientific, Waltham, MA, USA) for 72 h. Cell survival was assessed using the oxidation of Resazurin to the fluorescent Resorufin (Alamar blue dye, Cat. BUF012A, Bio-Rad, Hercules, CA, USA) following manufacturer’s instructions and published protocols [29]. In brief, Alamar blue was added to cells, they were incubated for 1–3 h, and survival was assessed in the form of fluorescence emitted by the live cells. Untreated or DMSO-treated cells were used as controls. Each experiment was repeated >3 times. Data analyses were performed using GraphPad Prism. Statistical significance and IC_50_ values were estimated using 2-way ANOVA and other analyses methods on GraphPad Prism. 

### 2.4. UV Exposure and Estimation of CPDs

For UV exposures, we used a “SOL-UV6 ultraviolet solar simulator” from Newport irradiators. It is equipped with a Xenon arc lamp where the output is varied using the integrated variable attenuator aperture, which provides the ability to vary the output from 10% to 100% of the maximum available solar constants. The output spectrum is flat from 320 nm to 400 nm and no values are below 300 nm. This is abbreviated as “ssUV” for “solar-simulated UV” throughout the text. Various cell types seeded at ~50% confluency, were treated with the AZ and analogs at concentrations mentioned in the data figures for 24–h. Cells at ~80–90% confluency (untreated controls), were washed twice with PBS and exposed to 18 kJ/m^2^ of ssUV in minimal amount of PBS (ex. 2 mL PBS in a 60 mm cell culture dish) without any dish lid in place. According to our previous research, AZ and its analogs do not absorb UV wavelengths (Supplementary Figure S1) and are more photostable compared to several well-known skincare product additives [24]. The ssUV apparatus was monitored almost daily using UV meters for any emissions below 300 nm. The dose was decided based upon the 80–90% cell survival post UV exposure. After exposure, one time point was collected immediately to assess iCPDs, whereas more dishes were incubated in cell culture medium, inside the cell culture incubator, for various time points to be collected post UV to assess dCPDs. For dCPD induction, it is important that the cell culture incubator is not opened for at least 30 min (the first time point post UV) to avoid possible interference from the CO_2_/O_2_ ratio, which will be altered upon opening the incubator doors. Cells were collected by scraping and washed once with PBS, which was followed by DNA isolation using a Quick DNA miniprep Kit (Cat. D3024, Zymo Research, Irvine, CA, USA). CPDs were assessed using our previously established DNA ELISA protocols [8]. In brief, ELISA was performed using mouse monoclonal Anti-CPD (Cat. CAC-NM-DND-001, Clone TDM-2, CosmoBio, Inc., Carlsbad, CA, USA). Flat-bottom, 96-well plates (Cat. 2801, Thermo Scientific, Waltham, MA, USA) were coated overnight with 50 µL of protamine sulphate (0.005–0.05%, Cat. P3369, Sigma, St. Lois, MO, USA) in distilled water. After washing twice with water, heat-denatured DNA was plated in triplicates from each sample, into the protamine sulphate coated plate wells (50–100 ng/well in 50 µL PBS) and incubated overnight at 37 °C. The plates were washed 5 times with 200 µL of PBST (PBS containing 0.05% Tween-20 Cat. P7949, Sigma), and blocked with 2–4% FBS in PBST (blocking buffer) for 1 h at room temperature (RT). The plates were incubated with 100 µL of a 1:1000 dilution of anti-CPD antibody in blocking buffer, and incubated for 30 min at 37 °C, which was followed by 5 washes in PBST. Next, the plates were incubated with 100 µL of a 1:2000 dilution of Biotin-F(ab’)2 fragment of anti-mouse IgG(H+L) (Cat. B-2763, Life Technologies) in blocking buffer for 30 min at 37 °C. Detection was conducted using 100 µL of a 1:10,000 dilution of Streptavidin HRP (Cat. 434323, Life Technologies) in 2% FBS for 30 min at 37 °C, which was followed by 5 washes. The plates were washed once with citrate phosphate buffer (24.3 mM citric acid monohydrate, J.T. Baker Cat. 0110-01- and 51.4-mM sodium phosphate dibasic, Sigma Cat. S7907, pH 5.0). The o-phenylene diamine dihydrochloride (Cat. P8287-50TAB, Sigma) was used as a HRP substrate at 400 µg/mL in citrate phosphate buffer for 5–10 min. A measure of 50 µL of 2M H_2_SO_4_ was used to stop the reaction as soon as the color developed (5–10 min incubation in substrate), and OD was measured at 490 nm using a Synergy-2 multimode microplate reader (BioTek, Winooski, VT, USA). 

### 2.5. Modulation of Amount of Melanin in Melanocytes

The amino acid tyrosine is essential for melanin synthesis, and it is added to the OptiMem medium at a proprietary concentration by ThermoFisher. Assuming this concentration to be “100% tyrosine”, we purchased the “custom-prepared, tyrosine-free” medium from ThermoFisher. This medium lacked tyrosine or its precursors, which include L-tyrosine, L-tyrosine-2Na-2H_2_O, L-tyrosine-Di-sodium salt, Hypoxanthine, and Sodium Hypoxanthine. Mel-a (pigmented mouse melanocyte) cells were cultured in regular cell culture medium containing 100% tyrosine, and in medium containing 20%, 4%, or 0% of the total tyrosine amount. This was achieved by diluting the regular OptiMem with the one containing no tyrosine (tyrosine-free, 0% tyrosine). Cells were cultured in these media for two weeks, and 200 µg/mL of MBPD or AZ was then added, and the mixture underwent a further culture for 2–3 weeks in the respective media. Since the cells cultured in 0%-tyrosine medium lost their pigment over time (Figure 5) and the cell pellets were almost white or opaque in color, we assumed that the minimal amount of tyrosine from the horse serum used in the cell culture medium was not sufficient for melanin synthesis. To estimate the amount of melanin, 3 × 10^6^–5 × 10^6^ cells were lysed in 1M sodium hydroxide (NaOH) by heating them at 100 °C for 1–2 h, which was followed by a 10-min spin at 14,000 rpm at room temperature to remove any cell debris. Absorbance of the supernatant was recorded using the supernatant in a 96-well plate in triplicates from each sample, at 460–490 nm. Synthetic melanin (Cat. M0418, Sigma) was dissolved in methanol and used as a positive control to generate standard curves to estimate the melanin amount in cell lysates. The number of cells in each pellet was used as a normalization factor for each experimental condition. In a complementary experiment, cells were first bleached out of melanin completely by culturing them in tyrosine-free medium, followed by 100/20/4/0% tyrosine (not shown) treatment. This seemed to be cytotoxic, comparatively more so in 100% tyrosine, probably owing to the toxicity of the sudden rush of melanin derivatives and the oxidative stress generated during melanin synthesis. 

### 2.6. Quantitative PCR (qPCR)-Based Assessment of Expression of Nucleotide Excision Repair (NER) Pathway Genes

Various cell types were collected 24-h post incubation with 200 µg/mL AZ and MBPD, with and without ssUV exposure. RNA was isolated using a Quick-RNA isolation kit (Cat. R1050, Zymo Research). The cDNA was prepared using a High-Capacity cDNA Reverse Transcription Kit (Cat. 4368814, ThermoFisher Scientific). The gene expression of *XPA*, *XPC*, and *MITF* genes was assessed using the following primers. β-actin was used as an internal control for normalizations and data analyses. F1-R1 and F2-R2 represents two primer pairs which were used in the qPCR experiments. 

XRCC1:

**F1:** TGGTGCTCAGTGGCTTCCAGAA, **R1:** TGGGAGTGTTGGCAAAGGCACA

**F2:** AATGGCGAGGACCCGTATGC, **R2:** CACGTAGCGGATGAGCCTCC

XPA:

**F1:** GAAGAACCCACGCCATTCACAG, **R1:** CTCGGTTTTCCTGCCTCACTTC

XPC:

**F1:** GGTATTGTCGTGGAGAAGCAGTC, **R1:** CACGGTTAGAGAAGCCTTTCACC

**F2:** ATTGCGTGCATACCTTGCAC, **R2:** TATCTCCTCAAACCCTGCTC

TYRP1:

**F1:** AGCCACAGGATGTCACTCAGTG, **R1:** GCAGGGTCATATTTTCCCGTGG

**F2:** CCAGAAAATTCTCACAGTCAGGAG, **R2:** CCATATCCAAGGCCCTGACA

MITF:

**F1:** GATCGACCTCTACAGCAACCAG, **R1:** GCTCTTGCTTCAGACTCTGTGG

**F2:** *AGCAAGAGCATTGGCTAAAGA,***R2:** *GCATGTCTGGATCATTTGACT*

DCT:

**F1:** GCAAGATTGCCTGTCTCTCCAG, **R1:** CTTGAGAGTCCAGTGTTCCGTC

**F2:** TTGAGAGGAGAGGAAAGGGC, **R2:** CACGCCATCCAAGGTCATGC

### 2.7. Immunofluorescence for Nitrotyrosine

Posttranslational modification nitrotyrosine was used as a marker of nitric oxide synthase (NOS) activity. Melanocytes were cultured on collagen-coated, glass-bottom dishes (cat. P35G-1.5-14-C, MatTek, Ashland, MA, USA). After various treatments, cells were fixed with 4% formaldehyde for 10 min at RT, washed with PBS, and permeabilized with 0.5% Triton-X in PBS for 10 min. After blocking for non-specific antibody binding using 5% goat serum (Cat. 16210064, ThermoFisher Scientific) and 1% globulin free BSA (Cat. 001-000-161, Jackson ImmunoResearch, West Grove, PA, USA) in 0.1% Triton-x in PBS (blocking buffer) for 2 h at room temperature, cells were incubated with 1:1000 dilution of anti-nitrotyrosine antibody (Cat. A-21285, ThermoFisher Scientific) overnight at 4 °C. Detection was conducted using Alexa Fluor 488 Goat Anti-rabbit IgG (Cat. A-11008, Life Technologies). Imaging was performed using a ZEISS Axio Observer microscope. Approximately 5–10 images per condition, with ~70–100 cells per image, were quantitated for nitrotyrosine fluorescence using ImageJ software.

## 3. Results

### 3.1. AZ and Its Analogs Do Not Show Any Cytotoxic Effects on Various Skin Cell Types

**Figure 2:**
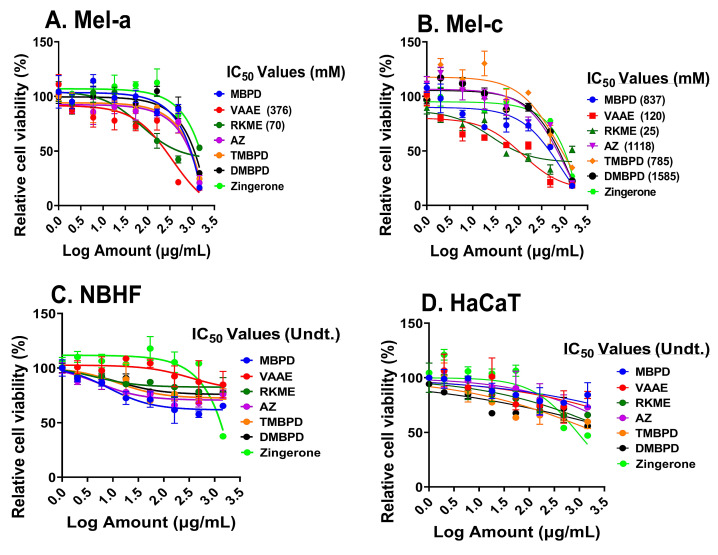
Assessment of cytotoxicity of AZ and its analogs. Various skin cell types, including pigmented (Mel-a) and isogenic albino (Mel-c) C57BL/6 mouse melanocytes in (**A**,**B**), respectively; newborn human fibroblasts (NBHFs, primary cells) in (**C)**; and human keratinocytic cell line, HaCaT, in (**D**) were incubated with AZ and its analogs for 72–96 h. Cell survival was tested in at least three biological repeats of each experiment using the oxidation of Resazurin to Resorufin (Alamar Blue dye) as a marker. Amounts of AZ and its analogs are mentioned in log (µg/mL), whereas IC_50_ values of each analog in Mel-a and Mel-c are mentioned in parenthesis in millimolar (mM). In Mel-a and Mel-c, most of the analogs showed no cytotoxicity or inhibition of cell growth only at very high concentrations. IC_50_ values for several analogs in (**A**,**B**) could not be calculated due to the non-cytotoxicity of the compounds, even at higher concentrations. All the analogs were comparatively non-toxic in the case of NBHFs and HaCaT, due to which the IC_50_ values could not be determined (undetermined [Undt.)]. Please see the Materials and Methods section for details on abbreviated names and details of the analogs.

The backbone structure of the phenolic alkanone is shown in Figure 1, whereas the chemical structures of Acetyl Zingerone (AZ) and its analogs, along with the abbreviations used in this manuscript, are described in the Materials and Methods section. Most of the analogs were non-cytotoxic at millimolar to molar concentrations, except for chemical toxicity at very high doses. This was true for isogenic, C57BL/6, pigmented (Mel-a), and albino (Mel-c) mouse melanocytes, newborn human skin fibroblasts (NBHFs), and human keratinocytes (HaCaT) (Figure 2), which constitutes all the cell types present in the skin. The origins of Mela-and Mel-c and the sources of all the cell types used are described in the Materials and Methods section. The 2-Vanillyl-acetoacetic acid ethyl ester (VAAE) and Raspberry Ketone Methyl Ether (RKME) analogs of AZ were the most toxic compared to other analogs (IC_50_ values ≥ 8 mM to molar) in few cell types used, including the pigmented, Mel-a melanocytes (Figure 2A,B). Since most of the skin cell types were unaffected even at extremely high levels (µg/mL) of the AZ analogs, cell survival assays demonstrated “non-sigmoidal” survival curves. The keratinocytes (HaCat cells) and newborn human fibroblasts (NBHFs, primary human skin fibroblasts isolated from foreskins) largely remained unaffected by these compounds such that the IC_50_ values could not be undetermined (Undt, in Figure 2C,D). Cell death at very high levels (>250 µg/mL) in all cell types was potentially due to chemical toxicity and not through cellular pathways altered by AZ analogs. 

### 3.2. AZ and Its Analog MBPD Significantly Inhibit CPD Generation

**Figure 3:**
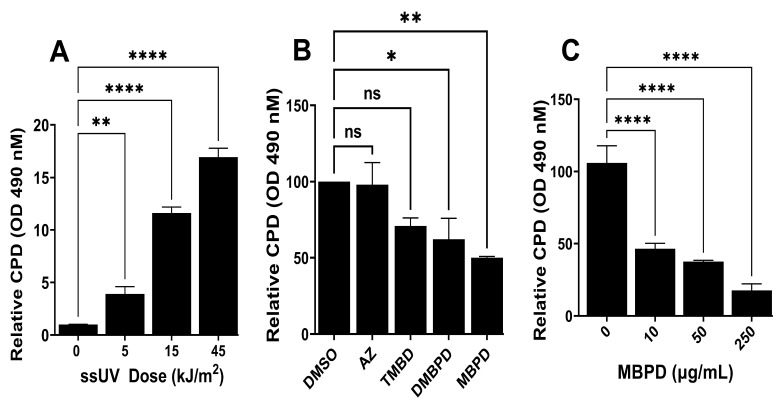
Inhibition of incident CPDs (iCPDs) by MBPD. (**A**) Detection of CPD generation in response to various doses and time points post exposure to sunlight-simulating UV (ssUV) in NBHF cells. The different doses of ssUV were 5 kJ/m^2^, 15 kJ/m^2^, and 45 kJ/m^2^. (**B**) NBHF cells were preincubated with 50 µg/mL of various analogs for 24 h, which was followed by exposure to 18 kJ/m^2^ of ssUV and assessment of iCPDs using ELISA. (**C**) NBHF cells incubated with different MBPD concentrations, which was followed by ssUV and iCPD assessment, as explained in (**B**). Each experiment was repeated at least three times with *p*-values of ≤0.01 (*), ≤0.005 (**) and ≤0.0001 (****). The “ns” is for “non-significant”.

CPDs were induced by using a “SOL-UV6 ultraviolet solar simulator (ssUV)” with a >95% output spectrum between 290 nm and 390 nm, as described in the Materials and Methods section. First, we tested the generation of “detectable” CPDs using various UV doses on NBHF cells, as shown in Figure 3A. The amount/level of CPDs is almost similar to the ones generated by UVA and narrowband UVB in our previously published studies [8,9,24]. This validated the effectivity of ssUV exposures in generating CPDs and the efficiency of the ELISA as an effective detection method. After testing various doses between 5 kJ/m^2^ and 45 kJ/m^2^, we selected 18 kJ/m^2^ of ssUV as a standard dose, which was relatively non-cytotoxic and decreased the viability of NBHFs by only 10–15% in 72–96 h post UV exposure but generated significant levels of iCPDs. The rationale was based upon our previous study where we selected UVA doses on the same principle and demonstrated the generation of detectable iCPDs and dCPDs [8]. Melanin chemiexcitation and the generation of dCPDs is not expected in NBHFs due to the lack of pigmentation, so the CPDs generated reflect those arising solely from direct exposure to ssUV (i.e., iCPDs). However, these cells also showed zero or minor repair of the iCPDs (not shown) for ~2 h, which has also been observed previously by our group [9]. Further, we have previously tested the inhibition of delayed or dark CPDs (dCPDs) by 25 µg/mL of AZ without any significant effect on the iCPDs [24]. Using this as a reference along with the IC_50_ values we calculated in Figure 2, we incubated NBHFs with 50 µg/mL of each analog for 24 h, which was followed by ssUV exposure and iCPD assessment with ELISA (Figure 3B). This assay suggested that while AZ is also partially effective, MBPD demonstrated a statistically significant blockade of ~50% of iCPDs in these cells. Although TMBPD and DMBPD both inhibited iCPD formation to varying extents, we observed maximum impact (Figure 3B) and minimal to no cytotoxicity (Figure 2) with MBPD. Therefore, we selected MBPD for further analyses in comparison with AZ in various cell types and experimental conditions. We further observed that MBPD can block iCPD formation in a dose-dependent manner in NBHFs, with significant iCPD reduction even at the lowest dose of 10 µg/mL (Figure 3C). 

**Figure 4:**
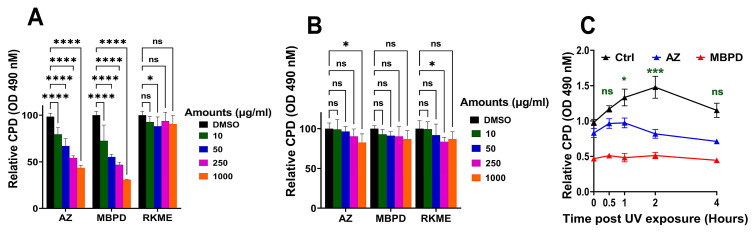
Inhibition of iCPD and dCPD formation by AZ and its analogues in mouse melanocytes. Isogenic pigmented (Mel-a) and albino (Mel-c) C57BL/6 melanocytes, in (**A**) and (**B**), respectively, were incubated with different amounts (µg/mL) of AZ, MBPD, and RKME for 24 h, which was followed by 18 kJ/m^2^ of ssUV and iCPD detection using ELISA. (**C**) Mel-a cells were preincubated for 24–36 h with 50 µg/mL of each of AZ and MBPD, then exposed to 18 kJ/m2 of ssUV. Cells were collected for iCPD detection immediately after UV exposure and incubated for various times post UV exposure in cell culture incubators, and then collected at respective times (0.5 to 4 h) for dCPD detection. CPDs were assessed using the DNA-ELISA approach. Each experiment was repeated at least three times with *p*-values of ≤0.01 (*), ≤0.001(***), and ≤0.0001 (****). The “ns” is for “non-significant”.

Next, we tested various doses of AZ and MBPD on different cell types for their efficacy in inhibiting the generation of iCPDs. This included human keratinocytes (HaCaT), pigmented C57BL/6 mouse melanocytes (Mel-a), and isogenic C57BL/6 albino melanocytes (Mel-c) (Figure 4 and Supplementary Figure S2). RKME was used as a negative control since it lacks pentane-2,4-dione as a functional group, which is a highly efficient scavenger of peroxynitrite (ONOO^−^). Accordingly, RKME should not affect CPDs, especially the dCPDs. Intriguingly, AZ and MBPD blocked iCPD generation in a dose-dependent manner, specifically in the pigmented C57BL/6 melanocytes (Mel-a) (Figure 4A) but not in the isogenic albino melanocytes (Mel-c) (Figure 4B). We constantly observed in multiple experiments that the efficiency of the iCPD blockade was still high for MBPD compared to AZ. For example, a 24-h preincubation with 50 µg/mL of AZ and MBPD blocked ~30% and 50%, respectively, of iCPDs in Mel-a cells. This was also true for longer preincubations with AZ and MBPD (up to 48 h), with the levels of each ranging from 50 to 100 µg/mL (not shown). However, the association of pigment or pigment-synthesis machinery with iCPD inhibition by AZ and MBPD remains to be explored empirically. Notably, RKME did not show any impact on iCPD in Mel-a or Mel-c, even at the highest dose of 1 mg/mL used in different assays.

Although AZ blocked ~50% of iCPDs in HaCat cells, this was observed only at 250 µg/mL, whereas MBPD and RKME did not show any effect (Supplementary Figure S2A). Higher amounts also led to the death of HaCat cells. While MBPD was ineffective in blocking iCPDs in HaCaT cells, it abrogated them in NBHFs in a dose-dependent manner (Figure 3C and Supplementary Figure S2B). In NBHFs, AZ blocked ~50% of iCPDs, but only at a very high dose of 1 mg/mL and had a minimal effect at other lower doses used in this study. We also noted that MBPD-mediated iCPD inhibition in NHBF was variable (Figure 3 vs. Supplementary Figure S2) but reproducible and statistically significant. Based upon the iCPD and cell viability data, we selected 50 µg/mL of MBPD and its parental analog AZ for a combined iCPD and dCPD assessment. MBPD inhibited iCPDs by ~50% and abrogated the delayed CPDs completely (Figure 4C). In comparison, while AZ inhibited dCPD formation almost completely, it was able to block iCPD generation by only ~30%. We previously demonstrated that ~50% of CPDs are generated by the chemiexcitation pathway at any given time point [8]. Integrating this with the 50% iCPD reduction and complete blockade of dCPDs, we extrapolate that MBPD can markedly inhibit ~70–80% of all the CPDs generated by UV, which is highly significant and has clinical implications. This also suggests the existence of dCPDs even before UV exposure, potentially due to known NOS hyperactivity in the pigmented melanocytes [8] and the presence of melanin pigment. Levels of these “pre-UV dCPDs” are reduced by MBPD-mediated inhibition of melanin synthesis, which reduces the amount of dCPDs formed by melanin chemiexcitation, in addition to MBPD-mediated upregulation of NER (described below). 

### 3.3. Underlying Mechanisms of AZ- and MBPD-Mediated Inhibition of iCPDs and dCPDs

MBPD and AZ inhibited the formation of iCPDs specifically in pigmented melanocytes, and both also inhibited the generation of dCPDs completely. Additionally, both AZ and MBPD had no significant impact on iCPDs in other cell types, including the isogenic, albino mouse melanocytes (Mel-c). To investigate the responsible mechanisms, we primarily focused on three mechanistic aspects. The first mechanistic aspect was the melanin pigment itself, since the AZ and MBPD-mediated effect on CPD inhibition was specific to the pigmented cells. Melanin is one of the indispensable raw materials for melanin chemiexcitation pathway [8] and the amino acid tyrosine is an absolute requirement for melanin synthesis [30,31]. We cultured the pigmented mouse melanocytes (Mel-a cells), in OptiMem base medium with the proprietary tyrosine concentrations added by the manufacturer (considered to be 100% tyrosine in this manuscript), and in medium containing different percentages of tyrosine generated by mixing the complete base medium with the custom-prepared, tyrosine-free OptiMem (20%, 4%, and 0%). Please see the Materials and Methods section for details on custom-prepared, tyrosine-free OptiMem and for its mixing with regular OptiMem to generate different tyrosine percentages.

**Figure 5:**
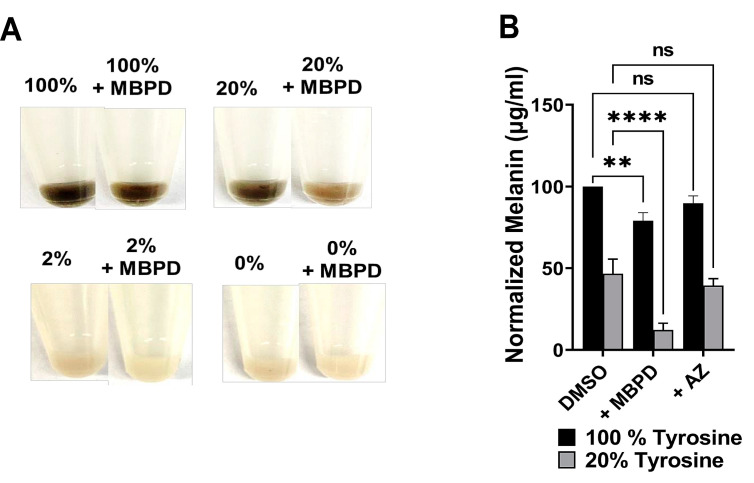
Inhibition of melanin synthesis by AZ and MBPD. Mel-a, the pigmented, C57BL/6 mouse melanocytes, were cultured in various amounts of tyrosine with 50 µg/mL of AZ and MBPD for ~2–3 weeks. (**A**) Representative cell pellets (~3 × 10^6^–5 × 10^6^ cells) are shown for each condition. The % numbers are % tyrosine amounts. Please see Methods for details. Proprietary tyrosine concentration from the manufacturer was considered to be 100% in the basal OptiMem media. The 20%, 4%, and 2%, and 0% tyrosine media were generated by mixing the basal OptiMem with the custom-made, tyrosine-free OptiMem medium. (**B**) Cell pellets were dissolved in 1M NaOH for ~1–2 h at 100 °C and the amount of melanin was quantitated using the spectrophotometric approaches described in the Materials and Methods section. Cells growing in 100% tyrosine were too saturated with melanin, whereas the ones in less than 20% Tyrosine were too light. Accordingly, 20% tyrosine was selected as an appropriate tyrosine amount for detectable differences of pigmentation in response to the incubation of Mel-a cells with AZ and MBPD. Normalization was performed using the cell numbers since longer incubations (2–3 weeks) of Mel-a cells with AZ and MBPD led to partial (10–15%) cell death. Each experiment was repeated at least three times with *p*-values of ≤0.005 (**), and ≤0.0001 (****). The “ns” is for “non-significant”.

**Figure 6:**
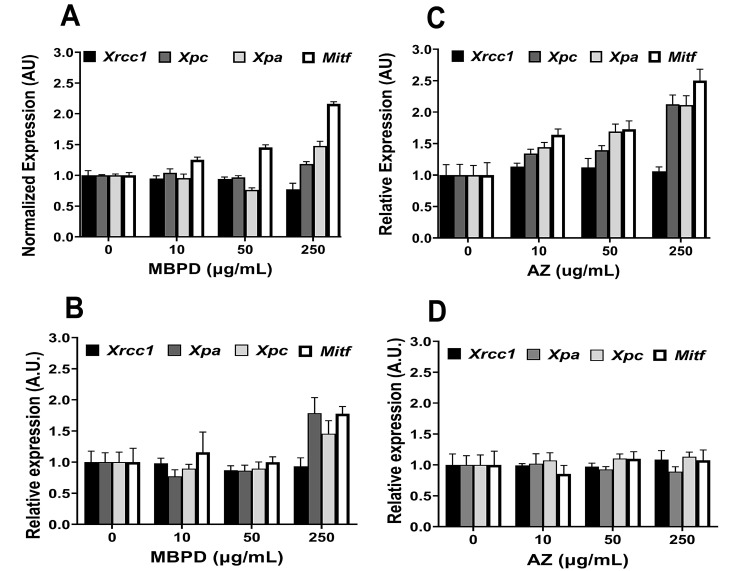
Upregulation of the nucleotide excision repair pathway by MBPD and AZ. Pigmented (Mel-a) and isogenic albino (Mel-c) melanocytes were incubated with AZ and MBPD for 24 h, which was followed by gene expression analysis using qPCR. MBPD upregulated the expression of *Xpa, Xpc*, and *Mitf* in both Mel-a (**A**) and Mel-c (**B**) cells, irrespective of pigmentation. Contrarily, the effects of AZ were specific to pigmented (Mel-a) cells (**C**) and the expression of these genes remained unaffected in albino (Mel-c) melanocytes (**D**). *Xrcc1*, a gene involved in the repair of double-stranded DNA breaks, remained unaffected by both AZ and MBPD. AU is arbitrary units of expression, where each dataset was normalized to the endogenous control (β-actin) and further with untreated cells to check the status of each gene. An average of at least three biological repeats is shown, with *p*-values of <0.005 for the highest dose of AZ and MBPD used in this experiment.

The tyrosine percentage was varied to manipulate the total amount of melanin into a range where pigmentation differences are detectable by spectrophotometry. Surprisingly, the cells co-incubated with 50 µg/mL MBPD showed ~25% reductions in melanin concentration when compared to the control group with 100% tyrosine. The inhibition of pigmentation by MBPD was observed more prominently (>50% reduction) when cells were cultured in 20% or less tyrosine (Figure 5A,B). However, AZ did not show any impact on melanin with different tyrosine variations. Since CPDs are primarily repaired by the nucleotide excision repair (NER), for the second mechanistic aspect, we checked the status of key genes of the NER pathway using qPCR analyses. A 24-h pretreatment of melanocytes with MBPD upregulated the gene expression of *Xpa* and *Xpc*, two principal components of the NER pathway [32,33] (Figure 6A,B) in both pigmented and albino mouse melanocytes. In addition, *Mitf*, which regulates NER positively [34], was also upregulated by MBPD. Interestingly, AZ also upregulated the NER pathway, but very specifically in the pigmented melanocytes and not in the isogenic, albino melanocytes (Figure 6C,D). Moreover, neither MBPD nor AZ had any impact on the genes involved in the base-excision repair pathway gene Xrcc*1*. We also tested the expression status of the above-mentioned genes at various time points post UV exposure in Mel-a melanocytes incubated with AZ and MBPD. As expected, UV exposure itself upregulated the NER pathway genes, which were elevated slightly in response to MBPD (Supplementary Figure S3) and AZ (data not shown) treatment. The third mechanistic aspect was that MBPD, and AZ seemed to reduce NOS activity, which is vital for melanin chemiexcitation.

**Figure 7:**
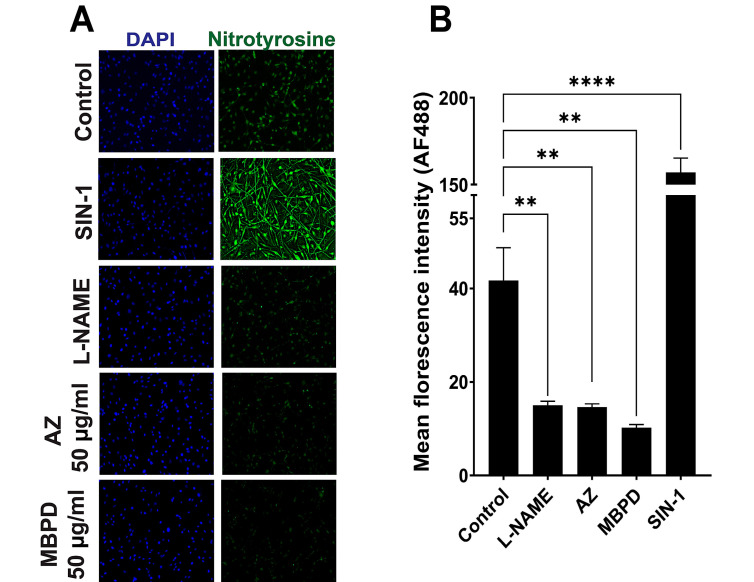
Effect of AZ and MBPD on nitric oxide synthase (NOS) activity and melanin chemiexcitation. Mel-a melanocytes were incubated with 50 µg/mL of AZ and MBPD for 24–36 h and the posttranslational modification called nitrotyrosine was assessed to be a marker of NOS activity. (**A**) Nitrotyrosine staining in response to AZ, MBPD, and the inhibition of nitric oxide synthase (NOSi) with N(ω)-nitro-L-arginine methyl ester (L-NAME). L-NAME inhibits all the isoforms of the NOS enzyme, which include eNOS, nNOS, and iNOS. Morpholinosydnonimine, commonly referred to as SIN-1, releases equimolar amounts of nitric oxide and superoxide, which cooperatively generate peroxynitrite that induces nitrotyrosine formation. SIN-1 was used as a positive control for nitrotyrosine detection. Cells were incubated with L-NAME for 48 h and with SIN1-1 for 4 h. (**B**) ImageJ-based relative quantitation of fluorescence intensity of Alexa fluor 488 (AF488) of the nitrotyrosine signal from (**A**). Image quantitation is a summary of three individual experiments with *p*-values of ≤0.005 (**), and ≤0.0001 (****). The “ns” is for “non-significant”.

We assessed nitrotyrosine, a posttranslational modification induced specifically by nitric oxide, as a marker of NOS activity using our previously established protocols [8]. While both AZ and MBPD reduced the amount of nitrotyrosine (and hence NOS activity), MBPD was comparatively more efficient (Figure 7A,B), suggesting that it is a potent NOS inhibitor or peroxynitrite scavenger. In conclusion, our findings suggest that AZ and MBPD are multifunctional phenolic alkanones which prevent iCPD and dCPD generation by: (a) inhibiting melanin synthesis and NOS activity, both of which are indispensable for melanin chemiexcitation; and (b) upregulating the NER pathway for a faster repair of CPDs. Further investigations are underway for detailed understanding of the molecular mechanisms behind the AZ- and MBPD-mediated inhibition of iCPDs and dCPDs. 

## 4. Discussion

UV exposure damages the DNA directly through CPDs and 6-4 photoproducts (6-4PP), and indirectly through oxidative DNA damage such as 8-Oxo-guanosine (8-oxoGua) [35]. Both types of damage lead to single-strand DNA breaks during the process of DNA repair. CPDs are the most predominant DNA lesions generated by various UV wavelengths [36] and are responsible for >80% of mutations, called “UV signature, C to T transition mutations” in sunlight induced melanoma [3,37]. Accordingly, effective blockade of CPD formation is the first line of defense against the damaging effects of UV exposure and can aid in the prevention of skin cancers. While sunscreens provide effective protection against erythema and iCPD formation, it is well recognized today that sunscreens can neither prevent the formation of sun-induced reactive oxygen species (ROS) within skin, nor protect against the formation of delayed CPDs (dCPDs) [38,39,40]. These deficiencies in the protective capacity of sunscreens, however, can be shored up with the inclusion of ingredients that separately provide the ability within skin either to attenuate excess ROS or block dCPD formation. 

In previous research, we documented the ability of AZ, with its unique chemical structure, to neutralize all the most reactive forms of ROS, including hydroxy- and peroxy- radicals, singlet oxygen, and peroxynitrite, with high efficiencies. We also observed significant attenuation of excess ROS in keratinocytes induced by UVA exposure, and the blockade of dCPD formation after AZ was added to melanocytes following exposure to UVA [24,25,26]. The ability to block dCPD formation was furthermore attributed to AZ’s capacity to scavenge peroxynitrite, reduce high-energy dioxetane intermediates on melanin via H**٠** atom donation, or function as a triplet energy acceptor for triplet melanin carbonyls (MCs), all of which could reduce the extent of dCPD formation from the melanin chemiexcitation (MeCh) pathway.

In this research, we further evaluated AZ, along with six of its structural analogs, for their effectiveness in blocking iCPD formation during direct exposure to UV in several different cell types, and dCPD formation in pigmented melanocytes. Regarding AZ’s ability to block dCPD formation, two important structure–activity relationships were revealed in these results. First, the replacement of AZ’s phenolic group with a methoxy group (MBPD), which prevents the possibility of H**٠**atom donation, did not impact the ability of MBPD to block dCPD formation (Figure 4C), suggesting that AZ’s efficacy does not arise from its ability to reduce dioxetane intermediates. Secondly, replacing the pentane-2,4-dione group with a ketone group (RMKE) completely abrogated the ability of the molecule to prevent CPD formation (Figure 4A), which suggests that maintaining the integrity of the pentane-2,4-dione group plays a central role in dCPD inhibition. Overall, these results support that the most likely explanations for AZ’s ability to inhibit the MeCh pathway stem from its ability to scavenge peroxynitrite or function as a triplet energy quencher for MCs. Interestingly, the energy quenching of triplet carbonyls by conventional antioxidant molecules has received considerable attention as a mechanism to prevent dCPD formation [41]. It would thus be interesting to evaluate AZ and MBPD within the same model system that was used to evaluate these other antioxidants to ascertain their triplet quenching capabilities.

The ability of AZ and several of its analogs to block iCPD formation was also a surprising discovery. Interestingly, all the analogs (TMBPD, DMBPD, and MBPD) that retained the structural integrity of pentane-2,4-dione group except AZ reduced iCPD formation to some extent in NBHF cells (Figure 3), with the highest efficacy displayed by MBPD. Indeed, a separate dose response study (Figure 3C) highlighted that a dose of just 10 ug/mL of MBPD inhibited iCPD formation by ~30–40%. These results indicate that these analogs operate through an energy transfer mechanism from an excited state of DNA following UV absorption to the analogs, which subsequently dissipate the excess energy as heat. This notion is further reinforced by the fact that AZ and its analogs display negligible absorption across the UV spectrum (Supplementary Figure S1), which rules out the possibility that they could be operating through a simple sunscreen-type effect. However, additional studies are required to elucidate the possibilities for energy transfer, including singlet–singlet as well as triplet–triplet energy transfers.

Since MBPD inhibited iCPDs and dCPDs maximally at considerably lower doses and inhibited melanin synthesis, we selected this analog for further analyses. MBPD inhibited both NOS activity (Figure 7) and melanin synthesis (Figure 5) to a higher extent than AZ, which had minimal effect on melanin synthesis. These results also indicate the MBPD-mediated inhibition of endogenous and UV-induced MeCh since NOS and melanin constitute two of the essential requirements for this pathway. This further suggests the existence of dCPDs in pigmented melanocytes even without UV exposure, due only to chronic, endogenous melanin chemiexcitation. The ~50% drop in iCPD formation at the 0H time point (Figure 4C) potentially arises from MBDP’s ability to impede endogenous dCPD formation from the MeCh pathway during the 24-h incubation period by inhibiting melanin synthesis, upregulating nucleotide excision repair (NER), or scavenging ONOO_¯,_ with the effects of NER upregulation possibly being magnified since there are now fewer “starting CPDs” to be repaired. However, based upon our previous research [8,9], we also predict that the amount of pre-UV CPDs should not be as high as 50% of all the CPDs generated by UV immediately after UV exposure at time 0H. Accordingly, a more detailed investigation is needed to completely understand the blockade of iCPDs by MBPD. Skin is neuronal in origin, and UV exposure also causes endocrine effects through nitric oxide [42,43]. However, a correlation between nitric oxide-mediated endocrine responses and iCPDs and dCPDs is beyond the scope of this manuscript. 

The upregulation of NER in both pigmented and albino melanocytes by MBPD, but only in pigmented cells by AZ, suggests a specific function and close association of AZ with the melanin synthesis pathway. It is known that DNA damage enhances melanin synthesis [44]; however, its correlation with CPDs is completely unknown. Our preliminary qPCR data, which need further validation, suggest the upregulation of two melanin synthesis pathway genes, *Dct* and *Tyrp1*, in the first 24 h of incubation with AZ and MBPD both (unpublished, not shown). This needs detailed investigation since the melanin estimation (Figure 5) was performed 3-6 weeks post incubation with MBPD and AZ, while the assessment of iCPDs and dCPDs, and *Dct* and *Tyrp1* expression, were performed within 24–48 h post incubation. We believe that the expressional upregulation of *Dct* and *Tyrp1* mRNA is short-lived and does not translate into the pigment melanin. In addition, melanin monomers are concentrated in perinuclear-coated vesicles during melanin synthesis [45]. These monomers are lipophilic and potentially able to enter the nucleus, suggesting the presence of melanin in the cytoplasm and nucleus, which is potentially carcinogenic owing to NOS-induced chemiexcitation [8]. Accordingly, we predict that MBPD inhibits processes associated with either tyrosine oxidation or the assembly of melanin monomers around the nucleus, which reflects lack of dCPDs within 48 h; whereas, visible downregulation of melanin synthesis occurs only when cells have undergone enough cell divisions to lose their remaining pigment. Further investigations are underway to reveal a correlation specifically in pigmented melanocytes between iCPD inhibition by MBPD and the melanin synthesis pathway. This also includes the investigation of iCPD inhibition by MBPD and AZ, and NER upregulation by MBPD in both the pigmented and isogenic albino melanocytes.

We are establishing cell culture-based methods to transfer the pigment melanin into keratinocytes. This includes “keratinocytes–melanocytes” co-culture, the incubation of keratinocytes with isolated melanosomes, or culturing cells in melanocytic-conditioned medium. This will assist in differentiating whether the iCPD inhibition by AZ and MBPD is associated with the melanin synthesis pathway, or if just the presence of the pigment melanin is sufficient for the downstream effect. Such functions will block MeCh and thus prevent CPD generation, leading to a significant reduction in the total amount of CPDs at any time point post UV exposure.

In conclusion, we propose that AZ and MBPD are extremely effective in protecting skin cells from the carcinogenic impacts of UV exposure by blocking the formation of CPDs. Based upon the experimental outcomes, we predict that that AZ and MBPD operate through two distinct mechanisms. Both are efficient ONOO^−^ scavengers and antioxidants and upregulate NER. However, MBPD is mostly operative through the inhibition of MeCh by inhibiting NOS and melanin synthesis, while AZ is mostly operative through NER upregulation and possibly triplet energy quenching. Detailed studies are underway to further elucidate the mechanisms responsible for CPD inhibition by both AZ and MBPD and several other AZ analogs.

In this regard, our research presented here showcases that AZ and its analog MBPD constitute two highly efficient compounds that not only inhibit the formation of dCPDs but also iCPDs. Since various cell types tolerated milli-molar (mM) concentrations of AZ and MBPD, we propose that AZ and MBPD appear to be safe and highly efficient “next-generation additives” to bolster the protection properties of sunscreens, cosmetics, or other specialized clinical skincare applications.

## 5. Conclusions

CPDs are one of the major carcinogenic DNA adducts generated by UV exposure and are responsible for >80% of mutations in sunlight-induced skin cancers. Blocking CPD generation is a prime preventive measure against UV-induced skin cancers, including melanoma. Existing sun blockers are short-lived, inefficient, and even photosensitizers, which might further enhance the carcinogenicity or other damaging effects of UV exposure. In this regard, we validated that AZ and MBPD are two plant-based phenolic alkanones which are highly efficient in blocking the UV-induced incident and delayed DNA damage. Further, these compounds are long-lived, non-toxic at high molar concentrations, efficient UV blockers, and also the blockers of carcinogenic DNA adducts. A detailed investigation is envisaged to further validate the association of these compounds with melanin synthesis and DNA damage/repair machinery. Additionally, studies using in vivo models, ex vivo human skin, or skin organoid cultures will establish AZ and MBPD as major constituents of “next-generation sunscreens”.

## Data Availability

No high-throughput data was generated in this manuscript and all the results/outcomes are presented either in the main figures or in the supplementary data.

## Supplementary Materials

The following supporting information can be downloaded at: https://www.mdpi.com/article/10.3390/antiox12020278/s1.
