# Supplementary material for: The Role of Acetyl Zingerone and Its Derivatives in Inhibiting UV-Induced, Incident, and Delayed Cyclobutane Pyrimidine Dimers
Source: Antioxidants (Basel). 2023 Jan 26;12(2):278. doi: 10.3390/antiox12020278 (PMC9952391; doi:10.3390/antiox12020278)
Supplement: Supplementary file 1 [file antioxidants-12-00278-s001.zip › antioxidants-2186361-supplementary.pdf]

Supplementary Data

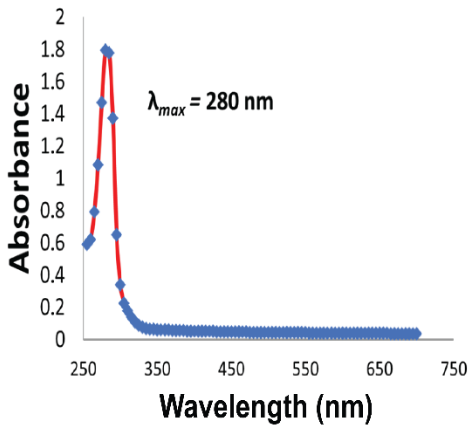

Supplementary Figure S1. UV-VIS absorption spectrum of Acetyl Zingerone. Maximum absorption was at 280 nm (UVC region). We used a sun-simulating lamp which emits >310 nm. Details of the instrument are mentioned in Methods section.

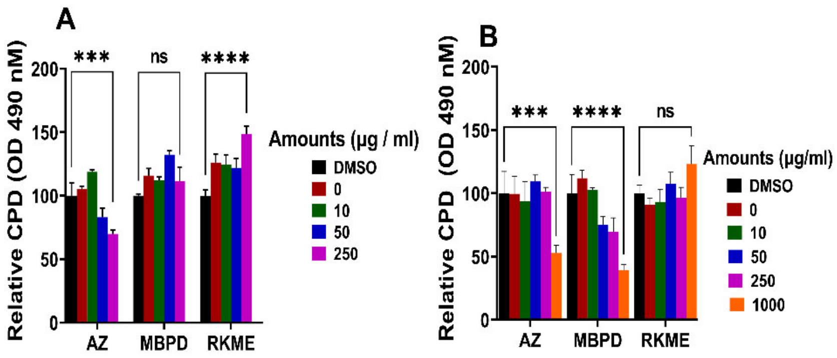

<u>Supplementary Figure S2. iCPD inhibition by AZ and its analogs in keratinocytes and skin fibroblasts</u>. Cells were pre-incubated with various amounts (µg/ml) of AZ, MBPD, and RKME for 24 hours followed by 18 kJ/m2 of ssUV exposure and CPD assessment using ELISA. AZ inhibited iCPDs in HaCaT cells **(A)** while inhibition was observed in NBHFs by AZ at very high doses and MBPD at moderate doses **(B).** The 1000 µg/ml of AZ was found cytotoxic to HaCaT cells probably due to chemical toxicity. Each experiment was repeated >3 times with p-Values ≤0.001(***) and ≤0.0001 (****). The “ns” is for “non-significant”.

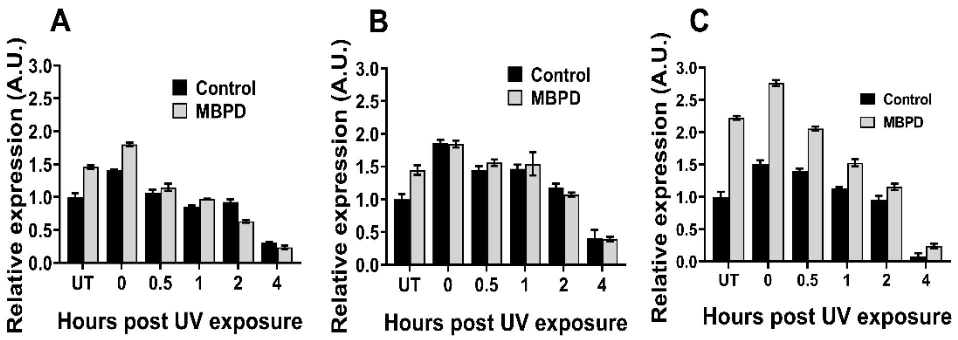

Supplementary Figure S3. Effect of MBPD on nucleotide excision repair pathway in response to UV exposure. Mel-a cells were incubated with 200 µg/ml of MBPD for 24 hours followed by 18 kJ/m^2 of ssUV exposure and cells were collected at various time points post UV exposure. RNA was isolated and expressional status of *Xpa* **(A)**, *Xpc* **(B)**, and *Mitf* **(C)** was assessed using qPCR. Each experiment was repeated >3 times with p-Values <0.01 to 0.0005 for comparisons between untreated (UT) and 0 to 0.5 hours post UV.
